# Molecular Biomarkers in IgE Immunoassays Used for Grass Pollen Allergy Diagnosis in European Clinical Settings

**DOI:** 10.3390/ijms27031393

**Published:** 2026-01-30

**Authors:** Lorena-Mihaela Gheorghita, Mariana Preda, Carmen-Saviana Marghidan, Miruna-Ioana Lazar, Ioana-Raluca Papacocea, Sylwia Smolinska, Florin-Dan Popescu

**Affiliations:** 1 Department of Physiology, Faculty of Medicine, Carol Davila University of Medicine and Pharmacy, 050474 Bucharest, Romania; lorena-mihaela.gheorghita@drd.umfcd.ro (L.-M.G.);; 2 Department of Allergology Nicolae Malaxa Clinical Hospital, Faculty of Medicine, Carol Davila University of Medicine and Pharmacy, 022441 Bucharest, Romania; mariana.preda@umfcd.ro (M.P.); florindanpopescu@allergist.com (F.-D.P.); 3 Allergology and Clinical Immunology Department, Nicolae Malaxa Clinical Hospital, 022441 Bucharest, Romania; carmensavianamarghidan@gmail.com (C.-S.M.); miruna-ioana.lazar@rez.umfcd.ro (M.-I.L.); 4 Department of Clinical Immunology, Faculty of Medicine, Wroclaw Medical University, 51-616 Wroclaw, Poland

**Keywords:** molecular diagnosis, biomarkers, grass pollen allergy

## Abstract

Grass pollen allergy has a high prevalence worldwide, making an accurate diagnosis critical in the framework of multifaceted environmental exposures. Our narrative review provides a comprehensive synopsis of component-resolved diagnosis biomarkers for pollen of Pooideae and Chloridoideae grasses, along with practical approaches in European clinical settings. We present a structured overview of allergen components utilized in singleplex, multiparameter, and multiplex IgE immunoassays. Molecular biomarkers have key roles in distinguishing genuine grass pollen sensitization from cross-reactivity and in assessing the risks associated with various IgE sensitization patterns, thereby enabling precise allergy diagnosis and facilitating targeted allergen immunotherapy. Diagnostic algorithms are provided to assist clinicians in making molecular biomarker-based personalized decisions. This tailored approach supports better management of patients with grass pollen sensitizations and allergies.

## 1. Introduction

The term ‘grasses’ is traditionally applied to herbaceous species belonging to the Poaceae family. This near-ubiquitous family of plants dominates and defines many ecosystems and is one of the few plant families found on all continents. Being among the most prominent plant families, grasses are not only central to terrestrial ecosystems but also play a crucial role in human livelihoods. The origins of this botanical family date back to the Late Cretaceous, and it now comprises almost 11,800 species in 791 genera, divided into 12 subfamilies. The most important subfamilies, in descending order based on the number of species, are Pooideae, Panicoideae, Bambusoidea, and Chloridoideae. Protein molecules from such pollen grains of temperate and subtropical grasses represent significant aeroallergens [1,2,3,4,5].

The Poaceae family (grasses) has two major evolutionary lineages around the globe: the BOP clade (acronym from initials of three subfamilies: Bambusoideae, Oryzoideae, Pooideae), including subfamilies that exclusively use the C3 photosynthetic pathway and dominate temperate and cooler regions, and the PACMAD clade (acronym from initials of six subfamilies: Panicoideae, Arundinoideae, Chloridoideae, Micrairoideae, Aristidoideae, Danthonioideae), including subfamilies that nearly all use C4 photosynthesis and dominate subtropical and tropical regions. Therefore, grasses of the Pooideae subfamily are typically considered temperate grasses. In contrast, grasses of the subfamilies Chloridoideae and Panicoideae are generally classified as subtropical (or tropical) due to their climatic adaptations and photosynthetic pathways [1,6].

Poaceae plants are found across almost all European ecosystems. Grasses thrive in diverse climates, including the humid-temperate climate of Western Europe, the humid-continental climate of Central and Eastern Europe, the Mediterranean climate of Southern Europe, and the boreal/subarctic climate of Northern Europe. Many grass species in the Pooideae subfamily, such as *Phleum pratense*, *Dactylis glomerata*, *Lolium perenne*, and *Poa pratensis*, dominate the European humid temperate and continental climate regions. In contrast, *Cynodon dactylon*, from the Chloridoideae subfamily, is spread throughout southern and Mediterranean regions, as well as warmer temperate and continental areas. Meanwhile, *Sorghum halepense* and *Paspalum notatum*, belonging to the Panicoideae subfamily, are generally less widespread, with limited distributions, especially in southern parts of Europe [6,7]. All grass species are wind-pollinated (anemophilous) and release airborne pollen grains during their flowering season. Life strategy traits associated with clinically significant high allergenic pollen include grasses with substantial abundance, the ability to produce relatively high pollen loads, quite long flowering periods, and good pollen dispersal. Grasses growing in Europe that fit this portrayal include genera in the temperate clade, such as *Phleum*, *Dactylis*, *Lolium*, *Poa*, *Anthoxanthum*, and *Holcus*, as well as the subtropical genus *Cynodon*. *Phleum pratense*, widely distributed in temperate climates, has the most relevant grass pollen aeroallergens in Europe and is among the best-characterized allergenic Pooideae grasses. A single inflorescence produces up to 1.2 million pollen grains over its flowering season, typically May to July in Europe [6,8].

The most abundant airborne grass pollen originates from tall meadow grasses such as *Phleum pratense*, *Dactylis glomerata*, and *Alopecurus pratensis* [9]. Wild Pooideae grasses from the Poeae tribe, such as *Phleum pratense*, and the Chloridoideae wild grass *Cynodon dactylon* have pollen grains with an average diameter of 30–35 µm. In contrast, Pooideae cereal grasses from the Triticeae tribe, despite their allergenic potential, do not disperse far due to their heavier and larger pollen with an average pollen grain diameter between 45 and 55 µm. The biometric threshold between Pooideae wild grasses and cultivated cereals is 45 μm. Moreover, maize pollen grains have diameters of around 90 μm, which is double the threshold. Thus, the allergenic contribution of cultivated cereals is significant only at a localized scale around agricultural areas but otherwise is weak [10,11,12,13].

An accurate diagnosis of grass pollen allergy is determined through both in vivo and in vitro allergy tests. In Europe, molecular diagnosis has emerged as a modern approach in clinical practice. To avoid ambiguity, it is essential to define molecular biomarkers as allergen components. These are either purified natural proteins or, more importantly, recombinant allergens that serve as species-specific markers or cross-reactive panallergens and may be classified as major or minor allergens. A major allergen is a defined molecule that binds specific IgE in at least about 50% of patients allergic to that source. Minor panallergens are molecules from the same pollen source that bind specific IgE in less than about 50% of allergic patients. It is critical to note that specific IgE indicates sensitization but does not always equate to clinically relevant allergy, the clinical context being crucial [1]. All such biomarkers used in IgE immunoassays are discussed in detail later in this article.

## 2. Overview of Grass Pollen Exposure and Allergy in Europe

In Europe, grass thrives in a wide variety of habitats, including grasslands, meadows, arable land, pastures, coastal areas, and urban and suburban areas such as lawns, parks, and roadside verges. Grass pollen currently ranks among the leading aeroallergens and is a main cause of IgE-mediated pollen allergy in most European countries. About 20% of the population is affected by grass pollen allergy, with noticeable variations between and within European countries [5,9,14]. Moreover, more than 40% of patients with allergic disease develop clinically relevant IgE sensitization to grass pollen [15,16].

Grass pollen is a significant cause of respiratory allergy in temperate regions, where grass pollen sensitization accounts for a substantial part of all IgE sensitizations to outdoor allergens [17]. Many species release their pollen in high concentrations during the pollen season, leading to allergic symptoms ranging from seasonal rhinitis/rhinoconjunctivitis to asthma in susceptible individuals. Grass-induced pollinosis is the most prevalent pollen allergy, affecting more than 95% of IgE-sensitized patients in Europe. Seasonal rhinoconjunctivitis is the most common clinical phenotype of grass pollen allergy, affecting millions of people and significantly impacting patients’ quality of life. Thus, pollen respiratory allergy poses a significant health and economic burden in Europe [5,18,19,20,21].

Isolated episodes and rare epidemic outbreaks of thunderstorm asthma have also been documented in Europe [22,23,24]. Case reports of contact urticaria resulting from lying down on grass and of anaphylaxis caused by direct exposure of abraded skin during play or sports on grass are worth mentioning [25,26,27,28]. Oral corn pollen hypersensitivity, manifesting as generalized urticaria, has only been sporadically reported after ingesting corn silk (*Stigma maydis*) infusion [29,30].

The allergy diagnosis of grass pollinosis typically begins with a detailed retrospective clinical history, followed by assessment of IgE sensitization with skin prick tests (SPT) and/or IgE immunoassays using whole allergen extracts (wae) from pollen and other screening aeroallergens to measure serum specific IgE (ssIgE) antibodies. The diagnosis based exclusively on anamnesis and on these in vivo and in vitro tests, which rely solely on wae, is still the most frequently used in Europe but often yields equivocal results. In European countries with high and prolonged exposure to various aeroallergen sources, a clinical decision support system including an additional electronic clinical diary (eDiary) and component-resolved diagnostics (CRD) as a strategy of precision allergy molecular diagnostic application is nowadays recommended to significantly improve the diagnostic accuracy and standardization in the clinical settings and to guide the optimal prescription of allergy immunotherapy (AIT), which depends on the precise identification of the triggering aeroallergen and represents the only disease-modifying treatment in eligible patients with allergic rhinitis/rhinoconjunctivitis to grass pollen [1,31,32,33].

Assessing the correlation between the symptom seasonality of grass pollen allergy and exposure to clinically significant aeroallergens is challenging in many regions of Europe due to interconnected factors. Pooideae grasses dominate in Europe due to their ecological adaptability to temperate climates and have a pollinating season from May to August in Central Europe, peaking in June. Grass species such as *Phleum pratense* and *Anthoxanthum odoratum* are less represented in Southern Europe than in Northern Europe. In higher latitudes, the grass pollen season begins later, whereas in Mediterranean regions, pollination lasts from March to July for a more extended period. Defining aerobiological thresholds provides a framework for identifying triggers of seasonal symptoms. For grasses, the pollen season commences on the first day of a sequence in which five out of seven consecutive days each exhibit ≥3 grains/m^3^, with a cumulative count of ≥30 grains/m^3^ across those five days; it ends on the last day meeting these criteria in a comparable sequence. Concentrations of at least 50 grains/m^3^ characterize high pollen days [34].

The pollination periods of different types of temperate grasses widely overlap in Europe. A sequential flowering has been observed, with *Bromus* and *Hordeum* species tending to flower in the early part of the season, followed by *Dactylis*, *Poa*, and *Festuca* species in the late-early to middle of the season, and *Phleum* flowering towards the latter half of the season. *Lolium* species have longer active flowering periods. Persistence and high mobility of grass pollen result in increasingly diverse seasonal pollen communities. Grass genera display discrete, temporally restricted peaks of pollen incidence that vary with latitude, revealing that the taxonomic composition of grass pollen exposure changes substantially across the allergy season. From an allergy perspective, such timings of pollen release are less significant, as all Pooideae grasses produce pollen with highly cross-reactive IgE sensitization [6,35].

The diagnosis of IgE sensitization to Poaceae pollen may become more complex as subtropical grass *Cynodon dactylon* spreads into warm-temperate climates where Pooideae grasses are more prevalent. In areas where multiple Poaceae subfamilies coexist in the same habitat, it is challenging to determine which grass species trigger symptoms in sensitized patients. The species share similar pollen grain characteristics and are morphologically indistinguishable [36,37,38,39]. *Cynodon dactylon* is widespread across Europe, particularly in southern and central regions with Mediterranean, temperate, and transitional climates. It is found as far as 50° N. It can contribute to late-season grass pollen in temperate Europe, typically after the peak of Poaceae grass pollen in May–June. This Chloridoideae grass’s main flowering period extends from July to September in the warmer temperate regions of continental Europe. In the UK, the flowering period is often later and shorter, lasting from August to October. In Mediterranean areas, it begins earlier and lasts longer, starting in May, being significant in June and July in Italy, and lasting until November in Malta [40,41,42,43,44]. Therefore, keep in mind that European patients with pollen allergy symptoms in the summer months (June to August) may be sensitized to *Cynodon dactylon* [36].

A relatively low degree of cross-reactivity has been reported between *Phleum pratense* and *Cynodon dactylon* pollen. Sensitization to Bermuda grass in patients from temperate regions with no residence history indicative of exposure to this Chloridoideae grass was reported, along with the genuine co-sensitization to Bermuda grass and Timothy grass. Moreover, monosensitization to *Cynodon dactylon*, although rare, has also been reported in both adults and children from temperate regions where *Cynodon dactylon* is present alongside Pooideae grasses. SPT and IgE immunoassays with pollen-wae are not sufficient to identify the primary sensitizing grass [45,46,47]. *Sorghum halepense* and *Paspalum notatum* are less common in Europe than *Cynodon dactylon* and exhibit IgE cross-reactivity, particularly to group 1 allergens, and their cross-reactivity with grasses of the genus Pooideae is limited. In semitropical regions, cross-reactivity between subtropical grasses is more pronounced. In contrast, in temperate regions, grasses of the genus Pooideae dominate sensitization and exhibit only limited cross-reactivity with subtropical grasses [1,48,49].

Co-exposure to grass and non-grass pollen, along with fungal spores, varies across Europe, thereby obscuring the accurate assessment of the relationship between grass pollen allergy symptoms and exposure to other triggering aeroallergens. In many European regions, the grass pollen season temporally overlaps at least partially with the pollination periods of other anemophilous plants, such as trees and weeds, complicating the diagnostic use of pollen calendars for allergists, particularly in polysensitized patients [50,51,52].

The grass pollen season partially overlaps with weed pollen (mugwort, ragweed) from late July to September in many parts of continental Europe. It also overlaps extensively with tree pollen (olive, plane) in Southern Europe, where the spectrum of allergen exposure is much broader, and many plants pollinate simultaneously. The co-occurrence of *Alternaria* spores, *Olea europaea*, and grass pollen is observed in the Mediterranean region in May and June. In Central and Western Europe, the overlap between grass pollen and spores of *Alternaria alternata* occurs mainly in June and July. *Alternaria*, *Cladosporium* spores, and Poaceae pollen may be present in the air simultaneously, primarily in humid temperate climates. In Central and Northern European regions, the pollination periods of Pooideae grasses and birch are well temporally separated but may exhibit partial temporal overlap in Southeastern European regions [1,7,19,53].

Along with co-exposure to other sensitizing non-grass pollen types and fungal spores, geographic area, altitude, key local plant airborne allergenic sources, grass species and their distribution, and the interannual and regional variability in pollen production, potency, and allergen content per pollen grain may also confound symptom attribution and reduce specificity in correlations. Molecular aerobiology, by determining aeroallergens in ambient air by ELISA, is a valuable supplementary tool to pollen counting. DNA metabarcoding has also been used to investigate the spatiotemporal composition of the grass pollen season, given its ability to distinguish individual taxa in community samples. Additionally, novel environmental DNA (eDNA) sampling and quantitative polymerase chain reaction (qPCR) have been employed to determine the relative abundances of airborne pollen from common grass species. Besides pollen counts, none of these new methods is currently used in clinical settings. Meteorological and weather-related factors, including temperature, humidity, precipitation, air currents, wind, and storms, influence pollen release, dispersal, concentration, and allergen potency, which may also lead to mismatches between exposure assessments and symptom timing or severity. Furthermore, air pollution may exacerbate symptoms, serving as another confounding factor alongside those related to pollen [1,6,8,54,55].

In addition to evaluating symptom seasonality, establishing clinical phenotypes based on the severity/frequency of allergy symptoms, as well as comorbidities, is critical for an accurate diagnosis, as detailed in Section 5 [19].

## 3. Molecular Grass Pollen Allergens Used as In Vitro Biomarkers

Key molecular grass pollen allergens used in singleplex, oligoplex, and multiplex IgE immunoassays for component-resolved diagnosis of Poaceae pollen allergy in Europe are β-expansins Phl p 1, Lol p 1, and Cyn d 1, expansin-like Phl p 2, berberine bridge enzyme-like Phl p 4, ribonuclease Phl p 5, C-terminal domain of Phl p 5-related Phl p 6, polcalcin Phl p 7, Ole e 1-like Phl p 11, and profilin Phl p 12. Most of these molecular allergens are recombinant (r) and a few are highly purified natural (n) components used in commercial IgE immunoassays for comprehensive and accurate IgE sensitization profiling in grass pollen allergy patients. Carbohydrate cross-reactive determinant (CCD) markers are often tested alongside to rule out false positives [31,56,57,58,59].

Group 1 and group 5 allergens are responsible for IgE sensitization with a prevalence exceeding 90%, and their relationship with allergic symptoms has been clearly demonstrated. Sensitization to group 1 is more prevalent, but group 5 usually induces higher ssIgE titers than group 1 [60,61,62,63,64].

**Group 1** grass pollen allergens, such as Phl p 1, Lol p 1, and Cyn d 1, belong to a divergent group of β-expansins (EXPBs) expressed at high levels in the pollen of grasses but not of other plant groups. They are highly conserved, sharing 60–70% sequence identity, and are present in all grass species, representing a specific biomarker of the Poaceae family. The vast majority of grass pollen-allergic individuals, often exceeding 90–95%, have specific IgE antibodies to group 1 grass pollen allergens. Phl p 1 from *Phleum pratense* and Lol p 1 from *Lolium perenne* are the most extensively studied allergens from the Pooideae grasses. Such acidic glycoproteins, representing major allergens of grass pollen, share an even higher sequence identity of 85% to 95% at the amino acid level with EXPBs from other Pooideae species. Group 1 grass pollen allergens with high sequence identities and homologies include, in addition to Phl p 1 and Lol p 1, other cross-reactive allergen components: Ant o 1 from *Anthoxanthum odoratum*, Dac g 1 from *Dactylis glomerata*, Hol l 1 from *Holcus lanatus*, Agr a 1 from *Agrostis alba*, and Poa p 1 from *Poa pratensis* pollen grains; therefore, they are extremely cross-reactive allergens in the majority of the Pooideae grass species of temperate regions. Phl p 1 exhibits instead only partial cross-reactivity with Cyn d 1 [1,56,65,66,67].

Cyn d 1 exhibits cross-reactivity with the grass group 1 Chloridoideae allergen Zoy m 1 from *Zoysia matrella* and potentially with other β-expansins from the Chloridoideae subfamily, such as Bou g 1 from the grama grass *Bouteloua gracilis* pollen, and the Panicoideae subfamily, such as *Sorghum halepense* (Johnson grass) Sor h 1 and *Urochloa mutica* (para grass) Uro m 1 [54,68,69].

The EXPB family of glycoproteins is specifically and abundantly expressed in grass pollen [61]. EXPBs are torpedo-shaped proteins with two domains: a *N*-terminal domain 1, which is a six-stranded double-psi β-barrel (DPBB) resembling the catalytic domain of glycoside hydrolase family GH45 but lacking hydrolytic activity, packed tightly next to a C-terminal domain 2 having a β-sandwich fold and classified as a carbohydrate-binding module CBM63, which facilitates binding to cell wall polysaccharides, particularly glucuronoarabinoxylan. EXPBs induce pH-dependent elongation of plant cell walls without detectable enzymatic activity and play a crucial role in facilitating pollen tube growth and penetration by loosening the maternal cell walls in the stigma and style [67,70,71,72,73]. Some studies suggested that EXPBs are non-proteolytic glycoproteins that loosen plant cell walls by disrupting noncovalent junctions between the matrix polysaccharide glucuronoarabinoxylan, which binds cellulose [74,75]. The primary biological role of EXPBs is thus to regulate the growth of plant cell walls, which plays a crucial role in pollen tube development and pollen germination. Their expression is tightly controlled and occurs at specific stages, with low levels in immature flowers and maximal activation during the final stages of pollen development. This precise timing underscores the significance of regulating EXPB concentrations in pollen extracts [76,77].

Molecular allergens from group 1 are localized in the grass pollen coat, in the mature pollen grain wall composed of an outer envelope called exine (or exospore) and an inner cellulosic membrane called intine (or endospore), and also in the cytoplasmic matrix between *p*-particles (polysaccharide particles) and cell organelles (such as mitochondria), but not on amyloplasts (starch granules) [8,65,78,79,80]. It is noteworthy that these allergens are released from the pollen grain during hydration [8].

Phl p 1 allergen has two isoforms, or variants, named Phl p 1.0101 and Phl p 1.0102. Phl p 1 contains multiple epitopes for both T cells (which drive Th2 responses) and IgE antibodies (which mediate immediate hypersensitivity). T cell epitopes in Phl p 1 span the protein, including both species-specific and cross-reactive sites with other group 1 allergens. IgE epitopes in Phl p 1 are largely conformational but include multiple linear ones. The majority of IgE epitopes cluster on the C-terminal portion in a structurally oriented manner. Recombinant Phl p 1 (rPhl p 1) is not glycosylated and is very similar to native Phl p 1 (nPhl p 1), sharing many of the IgE epitopes with natural group 1 grass allergens [16,79,81,82,83]. In an immunoinformatic epitope study on 95% purity, completely functional Phl p 1, eight B-cell epitopes in each of the two Phl p 1 isoforms were predicted [61]. Using a validated enzyme-linked immunosorbent assay (ELISA) sandwich method to quantify Phl p 1, the mean Phl p 1 content is about 29 μg of allergen/mg of lyophilized native extract [61].

According to the EAACI Molecular Allergology User’s Guide 2.0, Phl p 1 exhibits a high IgE sensitization prevalence, with a range of 83–95%; therefore, it is considered a major allergen. It shares approximately 90% sequence identity with allergens from other members of the Pooideae subfamily, leading to extensive IgE cross-reactivity. ssIgE to rPhl p 1 is an essential biomarker of primary Poaceae pollen sensitization. ssIgE to other major grass pollen allergens is infrequently observed in the absence of ssIgE to rPhl p 1. Therefore, the absence of ssIgE to rPhl p 1 does not exclude genuine sensitization to grass pollen, which might be due, in fewer cases, to isolated IgE sensitization to other major allergenic proteins, such as Phl p 5. CCD-free rPhl p 1 helps in lowering the number of allergens required for the appropriate diagnosis of grass pollen allergy [1,31,80,84].

The major allergen Phl p 1 is associated with positivity in about 80% of patients with ssIgE to Phl p wae. To reach around 90%, Phl p 1 should be combined with the other major allergen, Phl p 5, as recommended by a World Allergy Organization position paper [85]. The specific IgE response to *Phleum pratense* pollen typically develops sequentially, progressing from an initial monomolecular phase to an oligomolecular phase and eventually reaching a polymolecular phase, with multisensitization to distinct non-cross-reacting molecules. This process, known as molecular spreading, begins with Phl p 1 as the primary “initiator” molecule in over 75% of patients with grass pollen allergy. The immune response then commonly expands to include Phl p 4 and Phl p 5, followed by Phl p 2, Phl p 6, and Phl p 11, and later may involve Phl p 12 or Phl p 7. Even among the few individuals with grass pollen allergies who begin their sensitization process with other molecules, IgE antibodies against Phl p 1 are quickly produced. Natural Phl p 4, alongside Phl p 1, is also regarded as an early indicator of grass pollen allergy. The pathophysiological consequence of this phenomenon is that the longer the disease persists, the more extensive the range of IgE sensitization to various pollen molecules becomes [1,31,84].

It is important to note that molecular monosensitizations can occur, most commonly with Phl p 1 and Phl p 4 and less frequently with Phl p 5. IgE sensitizations exclusively to Phl p 11, Phl p 7, or Phl p 12 are also possible, although these are even rarer [1,86,87,88].

The ratio of ssIgE to rPhl p 1 to ssIgE to *Phleum pratense* whole allergen extract (ssIgE-Phl p wae) cannot be precisely defined due to variations in individual patient sensitization profiles, immunoassay methods, and environmental factors such as pollen exposure. Limited published data suggest that the median concentration of ssIgE to rPhl p 1 is about 35–40% of that of ssIgE-Phl p wae, although this varies considerably. It is helpful to emphasize the complexity and heterogeneity of IgE profiles rather than relying on a single ratio. Moreover, the median ssIgE values for both Phl p wae and rPhl p 1 are elevated during the pollen season compared to preseasonal values. In patients with grass pollen allergy from a temperate sub-continental climate European region, the mean ssIgE concentrations for rPhl p 1 are even six times higher during the grass pollen season (May to July) than during the off-season (November to January) [89,90]. Furthermore, ssIgE values for the Phl p wae are generally higher than those for single molecular allergens, such as Phl p 1, because the extract contains multiple allergen components. However, by summing the IgE reactivity of individual grass allergen molecules and epitopes while avoiding overlapping detections, ssIgE values two to eight times higher than those for Phl p wae were observed. This apparent discrepancy may be attributed to the limiting factors of the amount of allergenic and non-allergenic components in a wae, as well as the heterogeneous and sometimes diluted nature of such extracts. Thus, the lower ssIgE values for wae compared to the combined IgE values of individual allergen molecules can be explained by ssIgE antibodies to specific components present in low abundance in wae [85,91,92].

Cyn d 1 from *Cynodon dactylon* pollen was the first group 1 allergen from subtropical grasses to be characterized as a glycoprotein with high *N*-terminal sequence homology to the well-characterized group 1 allergen of *Lolium perenne*, Lol p 1 [75,93,94]. Cyn d 1 is to some extent immunologically distinct from Phl p 1 from timothy grass, and sequence homology and structural comparison between Cyn d 1 and Phl p 1 reveal only a small conserved amino acid sequence surface area between these two grass pollen species allergens [65,95,96]. From amino acid composition determination and immunoelectrophoretic comparison studies, the amount of Cyn d 1 in the wae from Cyn d pollen is estimated to be abundant, at approximately 15% wt/wt [54,94]. Cyn d 1 exists in 11 isoforms (Cyn d 1.0101, Cyn d 1.0102, Cyn d 1.0103, Cyn d 1.0104, Cyn d 1.0105, Cyn d 1.0106, Cyn d 1.0107, Cyn d 1.0201, Cyn d 1.0202, Cyn d 1.0203, Cyn d 1.0204), each differing in its isoelectric point and molecular mass [1,97]. The C-terminus of Cyn d 1 presents major and minor IgE epitopes. There are some distinct Cyn d 1 IgE epitopes compared to group 1 allergens from the Pooideae or Panicoideae grass pollen, while one T-cell epitope of Cyn d 1 is highly conserved and cross-reactive with dominant T-cell epitopes of Lol p 1 and Pas n 1 [83,98,99,100]. The challenge with nCyn d 1, a CCD-containing, naturally purified glycoprotein, lies in its glycosylation near the amino terminus, which can lead to positive IgE reactivity tests in individuals without exposure to *Cynodon dactylon* pollen. Such results require complex clinical interpretation, as they may reflect low-affinity cross-reactivity of IgE primarily specific to temperate grass pollen allergens or nonspecific, clinically irrelevant binding to CCD components. When ssIgE to nCyn d 1 yields a positive result, it is essential to determine the protein nature of IgE epitopes by confirming negative results for ssIgE to CCD markers [5,54,75,101]. IgE immunoassays using nCyn d 1 may show false positives due to CCDs. IgE antibodies to CCDs are found in 22% of European pollen-allergic children, mainly in association with an IgE response to grass pollen, but also in 11% of populations where grass is not a significant aeroallergen source, often induced by other pollen grains. Therefore, the introduction of recombinant allergens from subtropical Group 1 grass pollen, such as rCyn d 1, improves diagnostic accuracy [1,102,103].

The prevalence of IgE sensitization to Cyn d 1 is 76–100%; therefore, this molecule is considered a major allergen. The ssIgE to rCyn d 1 is a suitable biomarker for the assessment of primary sensitization to this Chloridoideae grass pollen. Monosensitization to Cyn d 1 or elevated ssIgE levels to rCyn d 1 over rPhl p 1 indicates genuine sensitization to *Cynodon dactylon*. These sensitization patterns may be observed in patients from several European regions with warmer-temperate climates [54,96,104]. Although molecular IgE profiles exhibit a high prevalence of ssIgE toward the Cyn d 1 in Southern European countries such as Portugal, central and southern Italy, Albania, and Greece, the majority of patients exhibit multisensitization with ssIgE toward ≥ 3 major allergenic molecules of grass pollen Phl p 1, Phl p 5, and Cyn d 1, olive Ole e 1 and cypress Cup a 1. In some European regions, such as northern Italy, patients exclusively sensitized to *Cynodon dactylon* appear to be very uncommon, while in large forest-steppe zones in Eastern Europe, monosensitization to Cyn d 1 is reported in up to 5% of cases. Bayesian modeling positions Cyn d 1 as a key or dominant allergen in the hierarchy of group I allergens from temperate grass pollen. In Ukraine, in patients sensitized to Cyn d 1 and Lol p 1, there is an almost 100% probability of sensitization to Phl p 1. In conclusion, monosensitization to Cyn d 1 in temperate regions of Europe may be influenced by climate change, which facilitates the spread of subtropical grasses into such areas [19,46,47,52,105].

Groups 2 and 3 of grass pollen allergens are major allergens with similar three-dimensional structures. Although they differ considerably in their amino acid sequences and their isoelectric points, they exhibit only a slightly higher immunological reactivity for Phl p 3 on the B-cell conformational epitopes [106]. The Phl p 2 and Phl p 3 molecular allergens are proteins with homology to the C-terminal domain of the β-expansin protein family. Domain 2 of EXPBs, consisting of β-sheets, exhibits an evolutionary relationship and about 40% sequence identity to group 3 grass pollen allergens [57,71,72]. Although group 2 allergens exhibit significant sequence identities with the C-terminal portions of group 1 grass pollen allergens, there is no relevant IgE cross-reactivity in grass pollen allergic patients [80]. Phl p 2 is a representative of the large family of cross-reacting Pooideae grass pollen allergens classified as grass allergens group 2/3 [107].

**Group 2** allergens exhibit high allergenic activity and react with IgE in 40% to 60% of patients with pollinosis, with varying prevalence across studies. The presence of ssIgE to Phl p 2 is observed in 55–65%, and in some studies up to 80% of European grass pollen-allergic patients [1,57,80]. Similarly, around 60% of grass pollen allergic patients display ssIgE against the CCD-free rPhl p 2 [108].

Phl p 2 is thus considered a major allergen, with ssIgE to Phl p 2 serving as a secondary biomarker for Poaceae pollen sensitization. During the process of molecular spreading, sensitization to Phl p 2 typically develops later than to Phl p 1 and Phl p 5 [109,110]. Because IgE-reactive group 2 allergens are expressed in pollen grains of the Pooideae subfamily of grasses, and a homologous molecule of Phl p 2 has been identified in *Sorghum halepense*, but not in *Cynodon dactylon* pollen grains. ssIgE to rPhl p 2 represents a biomarker for confirming genuine sensitization to non-Chloridoideae grass pollen [1,31,57,80]. Phl p 2 from *Phleum pratense* pollen is a 10- to 12-kD non-glycosylated protein exhibiting 85–90% sequence identity between Pooideae species. It has one isoallergen, Phl p 2.0101 [1,107]. Allergens from group 2 were quantified at 1172 ng per 100 µg of total grass pollen proteins [8].

Phl p 2 is mainly located in the interior of the pollen grain, such as in the cytoplasm and sometimes within ribosome-rich areas. It is a molecular allergen more concentrated in the grass sub-pollen particles, which originate from the rupture of *Phleum pratense* pollen grains by osmotic shock. Therefore, ssIgE to Phl p 2 is a candidate biomarker for identifying patients at risk of asthma attacks during thunderstorms in the pollen season [111,112]. Moreover, Phl p 2 may be a primary trigger for sensitivity to grasses in patients with *Alternaria alternata* allergy. Alt a 1, its major fungal allergen, regulates the sensitivity to Phl p 2 in such a way that the probability of simultaneous sensitization to these two molecules in polysensitized people is almost 75% [105].

**Group 3** allergens have approximately 60% sequence identity with those in group 2 and were traditionally assigned a separate group number; however, they share highly similar conformational epitopes with group 2 grass pollen allergens and exhibit cross-reactivity with them in patients with grass pollen allergies. Although Phl p 2 and Phl p 3 reveal moderate sequence identity and significantly different isoelectric points, their three-dimensional structures are highly similar. In fact, Phl p 2 and Phl p 3 allergens signify a family of cross-reactive allergens that can replace one another in diagnostic IgE immunoassays [106,113].

**Group 4** allergens are found in temperate and subtropical grasses, with up to 85% IgE reactivity in sensitized individuals. However, average concentrations of ssIgE to natural group 4 allergens are relatively low, and patients who react in vitro exclusively to group 4 allergens often do not display clinical symptoms of grass pollen allergy. The timothy grass pollen Phl p 4 is the representative molecule of the group 4 allergens. It shares over 50% sequence identity with group 4 allergens in other grasses, such as Cyn d 4, which also contains a covalently linked FAD cofactor. Although the group 4 allergens share high sequence similarity, structural analysis may reveal subtle differences that account for their potential cross-reactivity and species-specific conformational epitopes [1,80,114,115,116].

Phl p 4 is classified as a flavin adenine dinucleotide (FAD)-dependent oxidoreductase within the vanillyl alcohol oxidase (VAO) family, specifically a glucose-methanol-choline (GMC) oxidoreductase. It functions as a glucose dehydrogenase, oxidizing glucose using molecular oxygen as an electron acceptor and FAD as a covalently bound cofactor. By supporting the carbohydrate metabolism in pollen, this enzyme is likely providing energy or structural components for pollen tube growth. At the same time, its oxidative activity may also contribute to pollen viability under environmental stress [1,8,80,114,115].

Phl p 4 is a highly basic, tryptase-resistant, berberine bridge enzyme-like, CCD-bearing glycoprotein involved in the synthesis of alkaloids and located in the pollen grain coat, exine (outer wall), and cytoplasm, often associated with amyloplasts and polysaccharide particles. Its amount in the pollen extract was determined to be 0.2 µg/mg. GMC oxidoreductase is believed to play essential roles in the deposition and assembly of Ubisch bodies and sporopollenin, contributing to the patterning of Poaceae pollen exine. Group 4 homologues have also been found in pollen from unrelated plants, such as birch and mugwort, and in plant foods, such as apple, peanut, celeriac, and carrot. IgE cross-reactivity seems to be primarily due to the presence of CCDs, and the term panallergen may therefore not be fully applicable for this group of grass pollen allergens [1,8,80,115].

As a major allergen in *Phleum pratense* pollen, Phl p 4 binds IgE in 70–75% up to 82% of grass pollen-allergic individuals, making it a major allergen linked to allergic rhinitis, rhinoconjunctivitis, and asthma. In most commercially available immunoassays, Phl p 4 is still used in its native form, containing extremely high CCDs, which may lead to clinically irrelevant cross-reactivity. This explains why, in some epidemiological studies, IgE reactivity to nPhl p 4 is found in over 90% of patients with grass pollen allergy. However, when the recombinant form of the molecule is used in assays, about 50% of that positivity is no longer confirmed. rPhl p 4 is helpful in allergy diagnostics, as it shows enhanced IgE binding compared to the natural glycosylated form in some immunoassays. About a quarter of patients were found to be monosensitized to only one *Phleum pratense* molecule, the most frequently detected monomolecular sensitized component, Phl p 4, along with Phl p 1. Although considered a major grass pollen allergen, it is not typically used in grass pollen allergen immunotherapy, unlike Phl p 1 and Phl p 5. A subgroup of patients monosensitized to Phl p 4 has an 18% positivity rate on skin prick tests and presents mainly with seasonal urticaria [1,31,86,117]. Recently, ssIgE to Phl p 4, as well as to Phl p 1 and Phl p 5, has been identified as an early indicator of allergic rhinitis in children from Northern Europe. Phl p 4 can also serve as a biomarker of IgE sensitization to Bermuda grass pollen, as it shares sequence homology with Cyn d 4, the major allergen of Bermuda grass pollen, but this needs to be investigated in relevant patient populations [1,80,115,118].

**Group 5** allergens are significant major allergens of temperate grasses. Such homologous allergens are found in all grasses of the Pooideae subfamily but not in the pollen grains of *Zea mays* and *Cynodon dactylon*, members of the Panicoideae and Chloridoideae subfamilies, respectively. As group 5 allergens have not been found in subtropical grass pollen, ssIgE to rPhl p 5 is particularly indicative of IgE sensitization to Pooideae grass pollen [1,80].

Grass pollen in Europe releases about 2–2.5 pg of group 5 allergens per pollen grain, with a wide range from less than 1 pg to 9 pg, and a seven-fold variation between different years and regions. Phl p 5 allergen is most often detected in coarse particulate matter; after rainy events, grass pollen allergens are rarely detected in the atmosphere as free pollen grains. In high humidity, allergens are released from pollen grains under conditions similar to those of pollination. Significant atmospheric washes induced by rainfall are also observed, as reduced pollen allergen levels follow precipitation. During thunderstorms, pollen grains may rupture due to osmotic shock, releasing allergen-containing particles. Phl p 5, along with Phl p 2, is the molecular allergen more concentrated in grass sub-pollen particles and therefore represents a candidate allergen for identifying patients at risk of thunderstorm asthma attacks. The concentration of Phl p 5 in the *Phleum pratense* pollen grain ranges from 2.7 to 3.5 µg/mg in an unpolluted environment, considering that a single pollen grain has a mass reported between 11 and 26 ng and an average diameter between 30 and 35 µm [8,55,112,119,120].

Phl p 5 is typically localized on the surface of amyloplasts (starch granules), but also in the cytoplasmic matrix between *p*-particles and cell organelles (such as mitochondria), in the exine and the intine of the *Phleum pratense* pollen grain. It is a cytoplasmic ribonuclease, essential in the enzymatic degradation of RNA, with a broad IgE cross-reactivity with other group 5 allergens from the Pooideae subfamily [1,8,65,121,122].

There are two isoallergens and 16 isoforms (variants) of Phl p 5 that are currently recognized by WHO-IUIS. The isoforms of group 5 allergens can vary within and between species. According to published studies, the Phl p 5a and Phl p 5b isoforms are immunologically comparable. Phl p 5b possesses ribonuclease activity in its C-terminal domain and plays a vital role in defense mechanisms. rPhl p 5b does not contain any consensus site for *N*-linked glycosylation, although it has non-critical *O*-linked glycosylation sites. rPhl p 5b reveals quite a similarity with nPhl p 5b and also possesses homology to other Group 5 allergens. Phl p 5b is a key target of IgE antibodies among 80–95% of grass pollen allergic patients [1,8,56,65,121,122]. According to allergen and protein databases, Phl p 5b is the previous name of Phl p 5.0201 (WHO-IUIS official nomenclature). Similarly, Phl p 5.0101 is the recommended name for Phl p 5a. The important isoform rPhl p 5.0101, which exhibits broad cross-reactivity with group 5 allergens from various grass species, is used in the macroarray multiplex IgE immunoassay, while the rPhl p 5.0109 isoform, whose detailed characterization has established it as a European Pharmacopoeia reference standard for allergen products associated with *Phleum pratense* pollen. There is a dissociation of the major IgE and T-cell-reactive peptide domains in Phl p 5 [54,109,123,124]. Phl p 5 is considered a major allergen of temperate grass pollen, ssIgE to Phl p 5 being detected in about 50–95.5% of European grass pollen-allergic patients. Although ssIgE antibodies to rPhl p 5 usually appear later than those to rPhl p 1 during sensitization trajectories, their concentration rises rapidly in many patients to high levels, underscoring their contribution to patients’ symptoms. It is noteworthy to mention that Phl p 5 is rarely the only molecule inducing grass pollen IgE sensitization. Moreover, ssIgE to rPhl p 5 may have prognostic value for disease severity or for the likelihood of progression from allergic rhinitis to asthma, but this needs to be confirmed in well-designed studies [1,80,122].

**Group 6** allergens are only expressed in Pooideae grasses, mainly in *Phleum pratense* and *Poa pratensis*, but not in other plants, so they detect a genuine sensitization to grass pollen. Patients sensitized only to these allergens are likely sensitized to Pooideae pollen. Group 6 allergens have been shown to react with 60–70% of sera from grass pollen-allergic patients [80,125]. Phl p 6 is an acidic peptide with an alpha-helical folding, considered a major grass pollen allergen, specific for the Pooideae subfamily. Two isoforms, Phl p 6.0101 and Phl p 6.0201, are officially recognized. Phl p 6 is localized on *p*-particles (polysaccharide particles), which represent one of the two types of pollen cytoplasmic granules (PCGs). It displays 60% sequence identity to the C-terminal domain of Phl p 5, but its *N*-terminal domain is distinct from that of group 5 grass allergens. The percentage of grass pollen-allergic patients with IgE sensitization to Phl p 6 ranges from 44% to 75%. Because it highly cross-reacts with Phl p 5, the molecular biomarker rPhl p 6 adds little diagnostic information once ssIgE to rPhl p 5 has been documented. IgE sensitization to Phl p 6 has been associated with more complex sensitization profiles. Moreover, if patients are sensitized only to Phl p1, but without ssIgE to Phl p 5 and Phl p 6, they are likely to be sensitized to non-Pooideae grasses such as *Cynodon dactylon* or *Zea mays* [1,8,104,125].

**Group 7** grass pollen allergens are polcalcins, characterized by EF-hand motifs that bind calcium ions; therefore, they are considered EF-hand calcium-binding proteins. They are primarily expressed in the pollen of various plant species (trees, grasses, weeds) and play physiological roles in processes such as pollen tube growth. Polcalcin is highly conserved across species, with amino acid sequences sharing high sequence identity (60–90%) and exhibiting widespread IgE cross-reactivity across diverse plant species. However, unlike profilin, polcalcin is not present in plant foods or latex, and its expression seems to be restricted to pollen grains [1,31,126,127].

Phl p 7 is an acidic EF-hand, calcium-binding homodimeric protein, representing the highly cross-reactive polcalcin of *Phleum pratense* pollen. rPhl p 7 is used as a non-glycosylated polcalcin biomarker indicating a broad IgE sensitization against pollen from various more or less unrelated plant sources, such as Bermuda grass, trees, such as birch, alder, olive, juniper, and weeds, such as goosefoot, ragweed, mugwort; therefore, it is considered a pollen panallergen. The hierarchy of IgE cross-reactivity among key tree and grass pollen polcalcins follows the order rPhl p 7 > rAln g 4 > rJun o 4 > rBet v 3, highlighting the role of rPhl p 7 as a superior biomarker for polcalcin pansensitization in allergic rhinitis. Because of cross-reactive IgE epitopes in these allergens, patients with ssIgE to group 7 allergens may be at risk of symptoms upon exposure to pollen from many plant species. Phl p 7 is a minor allergen; only about 7–15% of grass pollen-sensitized patients present ssIgE to Phl p 7, usually many years after disease onset. If the patient has ssIgE antibodies to Phl p 7 but not to Phl p 1 and/or Phl p 5, it is likely that the primary sensitization is to another non-grass pollen. It was previously thought that ssIgE to Phl p 7 may be associated with more severe symptoms in grass pollen allergy, including a higher prevalence of asthma and a higher frequency of allergic comorbidities. Although IgE sensitization to rPhl p 7 is a biomarker of polysensitization, it has recently been considered with unknown clinical relevance for respiratory symptoms. Double positivity for polcalcin and profilin has been associated with true polysensitization and disease progression and, consequently, with a poor clinical prognosis. Moreover, although this relatively potent allergen can induce high concentrations of ssIgE antibodies, it is not a candidate for allergen immunotherapy [1,31,54,80,104].

Specific IgE testing to pollen polcalcins can be performed with members of this protein family. Allergenic 2 EF-hand polcalcin proteins include monomeric birch *Betula verrucosa* Bet v 4, alder *Alnus glutinosa* Aln g 4, and dimeric Phl p 7. ssIgE testing for pollen polcalcin biomarkers is currently performed with rPhl p 7, as well as rAln g 4 or rBet v 4 [1,31,127]. High concentrations of ssIgE against polcalcin rPhl p 7, a heat-stable calcium-binding protein likely to cross-react with Zea m 7 from maize pollen, were suggested as a potential risk factor for urticaria to corn silk (*Stigma maydis*) infusion ingestion (traditional herbal medicinal product) in a patient with grass pollen allergy [29,30].

**Group 11** grass pollen allergens have been purified as glycoproteins from pollen of *Lolium perenne* and *Phleum pratense* and are not regarded as major allergens. The IgE sensitization to Phl p 11 ranges from 32% to 43%. It is an acidic polypeptide with homology to the soybean tryptase inhibitor. In intact pollen grains, Phl p 11 is found in the cytoplasmic granule compartment, and air pollution facilitates grass pollen rupture and therefore its release. Cross-reactivity of nPhl p 11 is related to both its protein moiety and its glycosylated side chains. When using rPhl p 11, cross-reactivity is limited to group 11 of grass allergens. This molecule cross-reacts with similar proteins from other temperate and subtropical grass species, making it a suitable biomarker of IgE sensitization to group 11 allergens. Although it exhibits sequence identity of more than 50% with Ole e 1 and other Ole e 1-related allergens from grasses (corn), weeds (thistle), and trees (olive, ash, privet, plantain), clinically significant cross-reactivity is observed only with grass homologues. As a biomarker, rPhl p 11 is most often associated with sensitization to other grass pollen allergens and therefore with more complex IgE sensitization profiles [1,8,80,128].

**Group 12** grass pollen allergens are profilins, small cytosolic proteins ubiquitously expressed throughout the plant kingdom. They bind to monomeric actin and other proteins, thereby regulating actin polymerization dynamics during various cellular processes and signaling pathways. Profilins from higher plants are highly conserved, revealing amino acid sequence identities of over 75% even between members from distantly related organisms. Extensive cross-reactivity of ssIgE against profilins from different plant sources has been described. No substantial cross-reactivity between plant and mammalian profilins has been observed to date. Depending on pollen exposure, 5–50% of pollen-allergic patients are sensitized to profilin. As plant food allergens, profilins are heat- and digestion-labile, and up to 50% of sensitized patients may have food allergies, most often oral allergy syndrome. Therefore, they are considered allergen biomarkers for broad IgE sensitization to plant allergens; their clinical relevance is variable but potentially present. There are different profilins available for in vitro diagnosis. In general, any vegetable profilin can identify profilin-positive patients, except for the cypress *Cupressus sempervirens* and pellitory *Parietaria judaica* profilins. ssIgE testing for pollen profilin biomarkers can be performed with rPhl p 12, as well as birch *Betula verrucosa* rBet v 2 [1,30,31,80].

Phl p 12 is a member of the profilin family of plant panallergens, associated with an extended range of cross-reactivity with pollen, food of plant origin, and latex. It binds to actin filaments and participates in cytoskeletal regulation; as such, Phl p 12 is located in the cytoplasm of timothy grass pollen grains. Phl p 12 is a minor pollen allergen with three isoforms. Its IgE sensitization prevalence is about 15% and is almost always associated with sensitization to major grass pollen allergens, and it appears later in the molecular spreading process, reaching only moderate concentrations of ssIgE antibodies in a minority of patients. Therefore, IgE to Phl p 12 is generally associated with a higher atopic background and/or longer disease duration. Moreover, Phl p 12 is not an appropriate biomarker for AIT, as profilin IgE sensitization can affect its efficacy when not associated with sensitization to the grass pollen major allergens [1,30,90,117,129].

Although IgE sensitization to profilin Phl p 12 is often clinically irrelevant, it is considered a risk factor for pollen-associated food allergy to various non-thermally treated fresh vegetables and fruits that contain profilin (fruit-pollen syndrome). Clinical reactivity manifested as oral allergic syndrome (oropharyngeal contact urticaria) to raw tomatoes, Cucurbitaceae (melon, watermelon, cucumber, zucchini), Rosaceae (peach, apple, pear, apricot, cherry, almond), and/or citrus and other exotic (orange, banana, kiwifruit, litchi) fruits is typically associated with profilin hypersensitivity. In contrast, patients tolerate these foods when processed [1,30,129,130,131].

CCDs have epitopes on complex *N*-glycans (*N*-linked glycans) attached to glycoprotein allergens from pollen, plant foods, and insect venoms. Such structures frequently contain a core α-1,3-linked fucose on the proximal *N*-acetylglucosamine and/or a β-1,2-linked xylose on the core mannose, which are absent in mammalian glycoproteins. In IgE immunoassays using natural allergen extracts or native purified components, CCDs often cause cross-reactivity. This can lead to diagnostic interference, including false-positive or overestimated ssIgE levels, and nonspecific binding in solid-phase platforms that retain residual *N*-glycans. Anti-CCD ssIgE is found in 20–30% of patients with pollen allergy, especially those with multiple IgE sensitizations. However, ssIgE antibodies against plant CCDs lack established clinical relevance, as anti-CCD IgE antibodies generally do not trigger mediator release from mast cells or basophils in vivo [54,132,133,134,135].

Strategies to mitigate CCD interference include CRD with non-glycosylated recombinant allergens, immunoassay platforms without CCD-susceptible matrices, the use of soluble CCD inhibitors, and screening for anti-CCD ssIgE antibodies. Recombinant allergen proteins expressed in *Escherichia coli* are not recognized by CCD because bacteria lack posttranslational glycosylation. CCD inhibitors include semisynthetic blockers and MUXF3-based inhibitors, such as bromelain conjugated to human serum albumin. The MUXF3 (Ana c 2.0101) carbohydrate epitope (o214), a purified *N*-glycan from pineapple (*Ananas comosus*) bromelain (nAna c 2), can detect ssIgE to *N*-glycans from most pollen sources. This nMUXF3 sugar epitope from bromelain serves as a biomarker for CCD in IgE immunoassays. Human lactoferrin rHom s LF (o214), produced in genetically engineered rice and glycosylated with plant CCD, is another CCD biomarker. In summary, CCDs contain *N*-glycan structures that can cause IgE cross-reactivity in assays, complicating the interpretation of results. *O*-glycans do not have a similar influence [54,132,133,134,135].

The grass pollen polygalacturonase Phl p 13 is not available for IgE immunoassays. In contrast, other pollen allergens, such as pectate lyases, defensins, Bet v 1-like proteins, cyclophilins, isoflavone reductases, and non-specific lipid transfer proteins, which are available for molecular diagnosis, are not naturally present in grass pollen [1,136,137,138,139].

## 4. IgE Immunoassays Using Biomarkers for the Molecular Diagnosis of Grass Pollen Allergy in Clinical Practice

In addition to in vivo allergy tests, in vitro IgE immunoassays for grass pollen allergy use various formats, ranging from singleplex to multiplex platforms [133,140,141,142].

Singleplex IgE immunoassays, whether solid-phase or liquid-phase grass pollen allergen extracts (Table 1), may be used to determine ssIgE against natural pollen extracts from various grasses from the Pooideae subfamily, Poeae tribe: *Phleum pratense*, *Dactylis glomerata*, *Lolium perenne*, *Poa pratensis*, *Festuca elatior*, *Holcus lanatus*, *Alopecurus pratensis*, *Agrostis stolonifera*, *Anthoxanthum odoratum*, *Avena sativa*, *Phalaris aquatica*, Bromeae tribe: *Bromus inermis*, Triticeae tribe: *Triticum aestivum*, *Hordeum vulgare*, *Secale cereale*, *Elymus triticoides*, Chloridoideae subfamily: *Cynodon dactylon*, Arundinoideae subfamily: *Phragmites communis*, Panicoideae subfamily: *Paspalum notatum*, *Sorghum halepense*, *Zea mays*. FEIA may be used to determine ssIgE against four mixes containing 5 or 6 natural pollen extracts from different grass species: *Phleum pratense*, *Lolium perenne*, *Poa pratensis*, *Holcus lanatus*, *Anthoxanthum odoratum*, *Bromus inermis*, *Secale cereale*, *Cynodon dactylon*, *Phragmites communis*, *Paspalum notatum*, and *Sorghum halepense* [19,140,141].

Oligoplex/multiparameter line blot IgE immunoassay may be used to determine ssIgE against natural pollen extracts from *Phleum pratense*, *Dactylis glomerata*, *Anthoxanthum odoratum*, *Secale cereale*, and a grass mix 2 containing *Phleum pratense* and *Secale cereale*. In contrast, the multiplex macroarray IgE immunoassay determines, besides ssIgE to eight grass pollen molecular allergens, ssIgE against natural pollen extracts from *Secale cereale*, *Cynodon dactylon*, *Phragmites communis*, and *Paspalum notatum* [133,143,144,145,146].

**Table 1:** Whole natural allergen extracts from grass pollen, which are used for in vivo and/or in vitro allergy diagnosis in Europe.

Grass (Poaceae Family) Species	English Common Names
Pooideae subfamily	
Poeae tribe; PCG2 (Poeae type grasses):	
*Phleum pratense*	timothy, Timothy grass, herdsgrass, common catstail
*Poa pratensis*	smooth meadowgrass, June grass, birdgrass
*Dactylis glomerata*	orchard grass, cocksfoot, hardgrass
*Lolium perenne*	perennial ryegrass, English ryegrass
*Holcus lanatus*	common velvet grass, Yorkshire fog, meadow soft grass
*Festuca elatior*/*pratensis*	meadow fescue
*Festuca rubra*	red fescue, creeping fescue
*Alopecurus pratensis*	meadow foxtail, common foxtail grass
*Cynosurus cristatus*	crested dog-tail grass, crested dogstail
Poeae tribe; PCG1 (Aveneae type grasses):	
*Avena sativa*	cultivated oat
*Anthoxanthum odoratum*	sweet vernal grass, spring grass
*Agrostis stolonifera*	spreading bent, marsh bent, white bent
*Agrostis capillaris/tenuis*	common bent, colonial bentgrass, black couch
*Arrhenatherum elatius*	false oat, false oatgrass, ribbon grass, tall oatgrass
*Phalaris aquatica*	bulbous canary grass, hardinggrass
Bromeae tribe:	
*Bromus inermis*	smooth brome grass, Hungarian brome
Triticeae tribe:	
*Triticum aestivum*	cultivated wheat
*Secale cereale*	cultivated rye
*Elymus triticoides*	creeping wildrye, beardless wildrye
*Hordeum vulgare*	cultivated barley
Chloridoideae subfamily, Cynodonteae tribe:	
*Cynodon dactylon*	Bermuda grass, Bahama grass, dog’s-tooth grass
Panicoideae subfamily, Andropogoneae tribe:	
*Sorghum halepense*	Johnson grass, Aleppo millet grass, Aleppo grass
*Zea mays*	cultivated maize/corn
Panicoideae subfamily, Paniceae tribe:	
*Paspalum notatum*	Bahia grass, bahiagrass, water couch
Arundinoideae subfamily, Molinieae tribe:	
*Phragmites communis*/*australis*	common reed grass

Note: English common names according to the European and Mediterranean Plant Protection Organization (EPPO) database [146]; PCG, Poeae Chloroplast Group. Grass species are organized in this table according to a systematic taxonomic hierarchy, prioritizing phylogenetic relationships and allergenic relevance, as well as documented clinical importance in allergy diagnostic literature, IgE reactivity frequency, and European geographic distribution; such an approach being chosen instead of alphabetical order to enhance practical interpretation for allergists. Authorized grass pollen wae in Europe are mentioned in the Paul Ehrlich Institute database [140,141,142,147,148].

A summary of the molecular allergens found in Timothy grass and Bermuda grass, the two representatives of the most clinically impactful grass subfamilies in Europe, and used for in vitro diagnosis of grass pollen allergy, is presented in Table 2 below.

Current IgE immunoassays are essential in vitro tools for measuring ssIgE antibodies against molecular components of pollen allergens, including recombinant proteins and some purified natural molecules, as well as grass pollen natural whole extracts. They can be used to assess the IgE-mediated sensitization profile to Poaceae grass pollen. Immunoassays used to detect ssIgE to molecular components and pollen allergen extracts are either singleplex, oligoplex/multiparameter, or multiplex, depending on the number of allergen extracts and molecular components used [133].

The standard reference commercial singleplex in vitro method for ssIgE to individual natural grass pollen extracts and molecular pollen components (Table 3) uses solid-phase-coupled allergens, and it is represented by the fluorescence enzyme immunoassay (FEIA). It uses a solid-phase-coupled, encapsulated cellulose polymer allergen (ImmunoCAP^®^, Thermo Fisher Scientific Inc., Phadia AB, Uppsala, Sweden), and it is the currently used singleplex assay for measuring ssIgE to individual grass pollen allergens. Based on the sandwich fluoro-enzyme immunoassay method, it enables assessment of the patient’s allergic sensitization profile not only to natural pollen extracts but also at the molecular level. Allergens are covalently coupled to a hydrophilic carrier polymer, a cyanogen bromide-activated cellulose derivative with a large surface area for protein binding. This method uses β-galactosidase-labeled anti-human IgE antibodies and 4-methylumbelliferyl-β-galactoside as a fluorogenic substrate, incubated with the bound complex to produce the fluorescent 4-methylumbelliferone. The fluorescence measurement is performed with a fluorocounter. This FEIA quantitative method delivers accurate results. The detection limits are 0.10–100 kU_A_/L. In the ImmunoCAP system, 1 kU_A_/L specific IgE represents 0.994 kU/L total IgE and is equal to 2.4 ng/mL specific IgE. The conversion ratios have not been established with other immunoassay systems [133,140,143,144].

Historically, ssIgE concentrations of ≥0.35 kU_A_/L were classified as positive. It was suggested that the lowest detectable limit for the autoanalyzer-based IgE immunoassay should be 0.1 kU_A_/L. ssIgE levels between 0.1 and 0.35 kU_A_/L warrant cautious interpretation in the context of the patient’s clinical history and symptoms, as they may be relevant in some instances. At any concentration, it is critical to differentiate between IgE sensitization and clinical allergy. Elevated sIgE levels alone, in the absence of corresponding symptoms, do not suffice to establish a diagnosis of allergy [74].

A multiparameter line blot immunoassay (Euroline™; Euroimmun AG, Lübeck, Germany) may be used to measure ssIgE against several grass pollen allergen extracts and recombinant pollen allergen molecules simultaneously (Table 3). Allergens coupled to a nitrocellulose membrane in strip format as thin parallel lines in well-defined positions on the solid phase react with ssIgE in the sample, anti-IgE antibodies labeled with alkaline phosphatase are added to form a complex, and bound antibodies react with the NBT/BCIP substrate (NBT: 4-nitro blue tetrazolium chloride solution; BCIP: 5-bromo-4-chloro-3-indolyl-phosphate, 4-toluidine salt) for colorimetric detection. Line scanning on the strip is performed with a BlotScanner. If anti-CCD IgE antibodies are detected in serum, as indicated by a positive CCD marker band, the serum should be re-incubated with the anti-CCD absorbent [133,145,146].

ImmunoCAP^®^ ISAC E112i (ImmunoCAP^®^ ISAC™, Thermo Fisher Scientific Inc., Uppsala, Sweden) is a molecular diagnostic test (Table 4) that simultaneously detects IgE antibodies to 112 allergen components (molecular allergens, either purified native or recombinant proteins). This immuno-solid phase allergen chip is a semi-quantitative test, and results are reported in International Standard Units (ISUs). The operating range of 0.3–100 ISU for ssIgE (ISU-E) roughly corresponds to a concentration range of 0.3–100 kU_A_/L of ssIgE. Molecular components are immobilized in triplicate on a glass slide coated with amine-reactive preactivated polymer as the solid phase, with four fields (4 microarrays per slide) for four samples. These allergen molecules bind ssIgE in the sample, and anti-human IgE antibodies conjugated to a fluorophore detect the IgE-allergen complexes. Fluorescence measurements and image processing are performed using a fluorescence scanner and microarray image analysis software. ImmunoCAP^®^ ISAC E112i is a repeatable and reproducible in vitro diagnostic tool for the determination of ssIgE. In grass pollen allergy, both this immunofluorescent multiplex microarray and FEIA demonstrate high sensitivity and specificity, and diagnostic agreement is observed across both techniques. Thus, ImmunoCAP^®^ ISAC E112i and ImmunoCAP^®^ FEIA have similar diagnostic performance in grass pollen allergy when using the recommended cut-off points [133,149,150,151].

The ALEX^3^ Allergy Xplorer multiplex IgE immunoassay (ALEX^®^, MacroArray Diagnostics, Vienna, Austria) is an enzyme-linked immunosorbent assay (ELISA)-based IgE macroarray platform (Table 4) featuring the currently most comprehensive panel of recombinant and purified natural allergen components (n = 218) and natural allergen extracts (n = 82) on the same test, in total 300 specificities. This diagnostic test is used for the in vitro simultaneous detection of ssIgE quantitatively (in the range of 0.3–50 kU_A_/L) and total IgE (tIgE) quantitatively in the range of 2–1000 kU/L (semi-quantitative 1001–2500 kU/L). Allergen extracts or molecular allergens, coupled to activated nanoparticles (polystyrene nanospheres) while preserving epitope complexity (state-of-the-art nano-bead technology), are systematically deposited onto a nitrocellulose membrane solid phase in the form of a macroscopic array in a polystyrene cartridge-type chip. This multiplex test uses larger spots, readily discernible to the naked eye, and is referred to as a macroarray. The particle-bound allergens react with specific IgE from the sample, and anti-human IgE detection antibodies labeled with alkaline phosphatase are added to form a complex. The protocol integrates a, thereby increasing the specificity of the test results. By using a CCD blocker during serum incubation in the standard ALEX^3^ immunoassay procedure, the number of false-positive ssIgE results is reduced significantly. Then the NBT/BCIP substrate is added, and the antibody-bound enzyme converts it into a colored insoluble precipitate. Then, image acquisition and analysis are performed using either an automated or a manual system, with the Raptor analysis software [133,149,152,153,154].

The ALEX CCD inhibitor eliminated CCD-positive signals detected by ISAC in 88.5% of cases. Based on sensitization values of 0.3–14.9 ISU or kUA/L, there was good agreement between ISAC and ALEX. The addition of allergen extract tests and the greater number of molecular allergens in ALEX led to the detection of more sensitizations and broader molecular detection. In the range of <15 ISU or kU_A_/L, ALEX may be more effective in detecting sensitizations [155,156,157].

## 5. Integration of Molecular Biomarkers into Clinical Algorithms for Diagnosis and Management of Grass Pollen Allergy

### 5.1. Molecular Biomarkers in the Diagnosis of Grass Pollen Allergy in Clinical Practice

CRD with grass pollen allergen components, as a strategy of precision allergy molecular diagnostic application, is nowadays recommended for grass pollen allergy after careful assessment of clinical history and conventional in vivo and/or in vitro allergy testing with whole allergen extracts of grass pollen.

**Figure 1:**
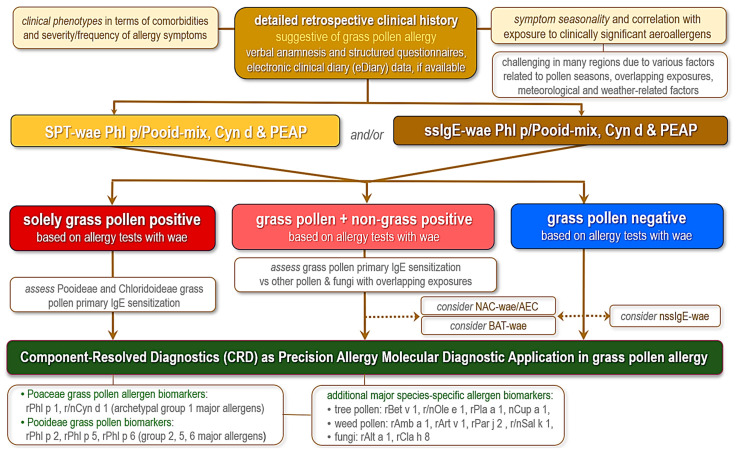
Diagnostic algorithm for grass pollen allergy with sequential integration of clinical evaluation, conventional testing, and recommendations for IgE immunoassays using molecular allergen biomarkers [32,52,54,104,118]. Note: CRD, component-resolved diagnostics; SPT, skin prick testing; ssIgE, specific IgE in serum (or plasma) sample; nssIgE, specific IgE in nasal secretions; wae, whole allergen extract(s); Pooid, Pooideae grasses; Phl p, *Phleum pratense*; Cyn d, *Cynodon dactylon*; PEAP, pan-European aeroallergen panel; NAC, nasal allergen challenge; BAT, basophil activation test; AEC, allergen exposure chamber; molecular allergen abbreviations according to the standardized nomenclature, consisting of the first three letters of the genus, followed by one letter from the species, and a numeral indicating the chronological order in which the allergen was identified/characterized within the source and/or the homology to allergens from another specie/s; r, recombinant; n, natural purified. This algorithm is an original interpretation with potential limitations. It cannot account for all individual parameters, circumstances, or potential decision pathways.

A proposed flowchart (Figure 1) outlines a diagnostic approach for suspected grass pollen-induced allergic rhinoconjunctivitis/rhinitis and/or asthma, aligned with current practices in allergology in clinical settings in Europe.

The initial clinical assessment includes a detailed retrospective clinical history as an essential foundation, with indispensable tools such as verbal anamnesis, structured questionnaires, and eDiary apps for symptom tracking. It is vital to evaluate symptom seasonality and correlate it with exposure to clinically significant aeroallergens, such as grass pollen, as well as to phenotypically characterize comorbidities and the severity/frequency of manifestations of rhinitis, conjunctivitis, and asthma exacerbations. Several challenges should be considered: variability due to overlapping pollen seasons, meteorological factors (e.g., temperature and humidity influencing pollen release), and weather-related factors. This step identifies patients suggestive of grass pollen allergy, guiding subsequent testing. Conventional diagnostic testing consists of SPT with wae and/or ssIgE to wae. Allergens tested include pollen extracts of *Phleum pratense* (Timothy grass) or Pooideae mix (representatives of temperate grasses), *Cynodon dactylon* (Bermuda grass), and PEAP (pan-European aeroallergen panel). This pan-European panel of commercial whole natural allergen extracts for SPT in suspected grass pollen allergy should include one grass species or mixed grass pollen, along with other pollen and fungi natural extracts, including birch, hazel, ash/olive, cedar/cypress and plane tree pollen, mugwort, ragweed, wall pellitory and prickly saltwort pollen, *Alternaria alternata* and *Cladosporium* spp., and house dust mites, and cat and dog epithelia [19,147,148,158,159].

Authorized whole grass pollen extracts for SPT in Europe [142] are available in various countries as individual extracts from the Pooideae representative *Phleum pratense*, a five-grass mix of extracts from Pooideae species: *Phleum pratense*, *Dactylis glomerata*, *Lolium perenne*, *Poa pratensis*, and *Anthoxanthum odoratum* or *Festuca elatior/pratensis*, and more extensive mixtures of grass pollen extracts from Pooideae species, including the previously mentioned ones along with additional wild grass species such as *Holcus lanatus*, *Arrhenatherum elatius*, *Alopecurus pratensis*, *Cynosurus cristatus*, *Agrostis stolonifera* or *Agrostis capillaris*, and *Bromus* spp. As grass pollen mixes for SPT currently available in Europe cover the most dominant Pooideae grasses, but not *Cynodon dactylon*, individual extracts from this representative of Chloridoideae are also available for SPT in many European countries. The natural extracts with grass pollen aeroallergens and those with allergens from grain cereals are distinct, despite belonging to the same botanical family (Poaceae) and exhibiting some structural similarities [19,147,148,158,159].

The algorithm based on the results of conventional in vivo and/or in vitro tests with wae (Figure 1) follows several branching paths. If grass pollen alone is positive (based on wae allergy tests), proceed directly to assess primary IgE sensitization between the Pooideae and Chloridoideae subfamilies using CRD. If grass pollen and non-grass tests are positive (based on wae tests), evaluate grass pollen primary IgE sensitization versus overlapping exposures (other pollens, fungi). If testing for grass pollen is negative (based on wae allergy tests), consider alternative diagnostic methods, such as nasal allergen challenge (NAC) with wae, allergen exposure chamber (AEC), or basophil activation test (BAT) with wae. Authorized natural wae for grass pollen nasal and bronchial allergen challenges in Europe [142] are available in some European countries. Consider BAT by grass pollen as an alternative to NAC [142,160].

AEC is a specialized medical facility designed to expose individuals to aeroallergens at precise, consistent concentrations in a controlled environment. AECs are advanced medical installations for standardized and reproducible allergen challenges. They simulate real-world aeroallergen natural exposure while ensuring strict control of allergen concentrations and environmental variables. Recently validated AECs are a specific, safe, and reproducible tool for the diagnosis of grass pollen allergy. Furthermore, due to their strong correlation with field testing, AEC challenges enable a more accurate assessment than NAC in patients with *Phleum pratense* pollen-induced allergic rhinitis [161,162,163].

While some published studies suggested that specific IgE in nasal secretions (nssIgE) against aeroallergens might be nonspecific, as it can be detected in both nonallergic rhinitis and healthy individuals, recent research suggests that nssIgE can be used as an additional diagnostic criterion for local allergic rhinitis (LAR). A key feature of LAR is the presence of nssIgE in 20–40% of affected individuals. When nasal cytology shows a significant accumulation of eosinophils in the nasal mucosa but no ssIgE is detectable, local nssIgE levels can serve as an alternative to NAC to support the diagnosis of LAR [164,165,166]. The presence of nssIgE antibodies specific to *Phleum pratense* pollen can be measured using the ImmunoCAP^®^ FEIA system (Thermo Fisher Scientific Inc., Phadia AB, Uppsala, Sweden), which utilizes encapsulated, solid-phase-coupled cellulose polymer allergens. Nasal secretions can be obtained either by inserting a sponge into each nostril or by bilateral nasal irrigation with normal saline. The detection limit for nssIgE is 0.35 kUA/L. Measurements are performed at baseline, 15 min, and 1 h after NAC [146,167,168].

**Figure 2:**
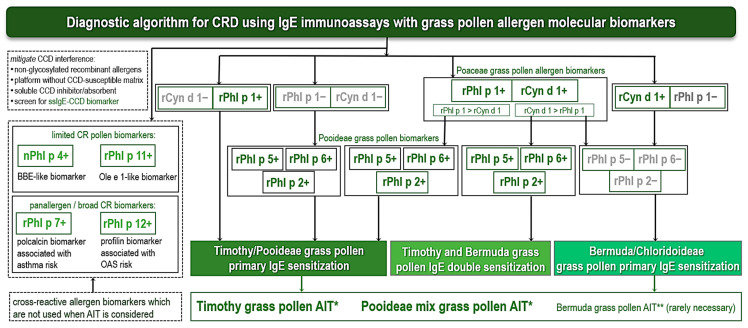
Diagnostic algorithm for CRD using molecular allergen biomarkers in IgE immunoassays for presumed grass pollen allergy in clinical settings in Europe [1,32,52,54,104]. Note: CRD, component-resolved diagnostics; CR, cross-reactive; CCD, cross-reactive carbohydrate determinants; BBE, berberine bridge enzyme; ssIgE, serum specific IgE; OAS, oral allergy syndrome; molecular allergen abbreviations according to the standardized nomenclature, consisting of the first three letters of the genus, followed by one letter from the species, and a numeral indicating the chronological order in which the allergen was identified/characterized within the source; r, recombinant; n, natural purified; AIT, allergen immunotherapy; * European Medicines Agency (EMA)-authorized sublingual immunotherapy (SLIT) products and nationally authorized sublingual and subcutaneous immunotherapy (SCIT) products; ** named-patient products (NPPs) typically as liquid solutions in metered-dose devices, without EMA authorization. This algorithm is an original interpretation with potential limitations. It cannot account for all individual parameters, circumstances, or potential decision pathways to consider when diagnosing grass pollen allergy.

A subsequent proposed algorithm (Figure 2) represents a comprehensive, stepwise framework for modern allergology, enhancing diagnostic accuracy, reducing misclassification, and facilitating targeted therapeutic interventions for grass pollen allergy in clinical settings across Europe. Application of CRD as a precision molecular diagnostic approach uses key Poaceae grass pollen allergen biomarkers: rPhl p 1 and rCyn d 1 as archetypal group 1 major allergens (β-expansins), rPhl p 1 being a marker for genuine sensitization to Pooideae (temperate grasses) subfamily pollen, while n/rCyn d 1 being a marker for Chloridoideae (subtropical grasses) subfamily pollen. Pooideae grass pollen biomarkers are rPhl p 2, rPhl p 5, rPhl p 6 (group 2, 5, 6 major allergens; highly specific to Pooideae, absent/minimal in Chloridoideae grasses). Additional major species-specific allergen biomarkers related to non-grass pollen belong to tree pollen: rBet v 1 (birch), rOle e 1 (olive), rPla a 1 (plane), nCup a 1 (cypress), weed pollen: rAmb a 1 (ragweed), rArt v 1 (mugwort), rPar j 2 (pellitory), and fungi: rAlt a 1, rCla h 8 (molds). CRD enables discrimination of genuine primary sensitization (e.g., rPhl p 1/5+ indicates Pooideae; rCyn d 1 dominant with rPhl p 5- indicates Chloridoideae). Cross-reactive panallergens such as plant profilin rPhl p 12, pollen polcalcin rPhl p 7 are often linked to polysensitization. Irrelevant cross-reactivity may be due to CCD. There is a scientific rationale and clinical utility for this CRD algorithm, given cross-reactivity considerations, high within Pooideae (Timothy extract representative), and limited between Pooideae and Chloridoideae/Panicoideae. CRD overcomes limitations of wae due to CCD interference or poor standardization. Diagnostic precision is reflected in improved identification of the primary sensitizing source, which is important for AIT optimization.

Grass pollen allergy is a typical example of efficient use of ssIgE to molecular allergens as biomarkers, for etiological confirmation of the presumptive diagnosis, for a comprehensive and accurate IgE sensitization profiling, distinguishing genuine IgE sensitization from sensitization due to cross-reactivity in multisensitized patients, providing a clear distinction between co-sensitization and cross-sensitization, for decreasing the need for provocation testing, and optimal decision making for treatment, including improving interventions to reduce allergen exposure, for more optimal pharmacologic treatment (choice, timing, and duration) tailored to the patient’s symptoms and pollen season, and for precise guiding of AIT prescription, choice of preparation, initiation and scheduling. The valuable role of CRD is obvious, particularly in European regions where polysensitization to airborne allergens is frequent [1,62,80,143,169]. Regarding additional value of CRD in grass pollen allergy, besides the fact that European climate changes with warming trends and increased human exposure to subtropical grass pollen allergens, population mobility introduces additional complexities in different grass pollen exposures: European individuals occupationally active in geographically diverse global regions encounter novel grass taxa, intra-European migration, such as between Mediterranean and northern/central zones (or vice versa) exposes sensitized patients to divergent airborne pollen profiles, and immigrants to Europe from subtropical/tropical regions (e.g., Africa, India, South Asia) may exhibit sensitization to different grasses [48,170,171,172,173].

Grass pollen is the most frequent inducer of IgE antibodies to classical CCD. Group 1 and group 4 allergen molecules are glycosylated, so the question of which of the two (or both) is contributing to the induction of IgE anti-CCD remains open. They hinder the assessment of results from in vitro immunoassays using wae and native allergen glycoproteins. This interference is most pronounced with high anti-CCD ssIgE levels (>7 kU_A_/L), and false positives primarily affect low-positive results (0.35–3 kU_A_/L). Modern IgE immunoassays use recombinant allergens produced in expression systems to be CCD-free. The use of *E. coli* recombinant proteins avoids the reactivity of *N*-glycan side chains. However, IgE reactivity could be partly influenced negatively by the absence of certain relevant post-translational modifications. Additionally, it should be noted that certain immunoassays use a cellulose matrix as a solid-phase carrier, which contains plant-derived *N*-glycan structures. In contrast, others use an integrated potent soluble CCD inhibitor/absorbent. The patient’s serum must therefore be screened for ssIgE to CCD [174,175,176,177,178].

### 5.2. Impact of Molecular Biomarkers on Allergen Immunotherapy for Grass Pollen Allergy

Regarding therapeutic decision-making, Pooideae AIT is highly effective in patients with grass allergy sensitized to temperate grass pollen, such as timothy or ryegrass. However, it should be mentioned that the clinical response depends on CRD demonstrating genuine sensitization to this type of pollen and adequate representation of the sensitizing allergens in the AIT products. These should not rely solely on allergenic cross-reactivity between the grass pollen species involved in sensitization but should also consider the epitope specificity of the different species [33,52,178]. Identification of the precise Pooideae grass species that induce primary sensitization is not essential; both mixed grass pollen extracts and single-representative ones, such as *Phleum pratense* pollen, are effective. Timothy grass allergens are sufficient in providing immunity to other temperate grasses due to extensive cross-reactivity with other Pooideae species allergens [178,179,180]. Patients exposed and sensitized to temperate grass pollen should be treated with Pooideae AIT products, while patients primarily sensitized to subtropical Chloridoideae grass pollen would be likely to benefit from AIT optimized to contain Bermuda grass pollen allergens. Chloridoideae pollen lacks group 5 allergens and exhibits subfamily-specific epitopes, particularly in group 1 allergens. Thus, Pooideae AIT may not be effective in patients monosensitized to *Cynodon dactylon*, and it could, in theory, induce new group 5 sensitizations [48,181,182]. A five Pooideae grass AIT product displays different IgE epitope repertoires, may generally better cover the sensitization profiles, and therefore is expected to elicit a broader spectrum of blocking antibodies in patients from Southern Europe, who, in principle, are less exposed to pollen from Timothy grass than from other grasses and may present double sensitization to Pooideae and Chloridoideae grass species. However, an AIT product with the one grass species represented by *Phleum pratense*, containing all important allergen groups, is as effective as an AIT with several related grass pollen species in temperate regions [6,33,52,80].

It is important to underline in such circumstances that there is currently no evidence of clinical superiority based on the number of grass species included in the grass pollen AIT product composition. Moreover, another significant aspect to mention is that children and adults with primary allergy to temperate grass pollen in temperate regions show similar five-grass-pollen SLIT efficacy, irrespective of polysensitization to subtropical grass pollen or geographical location. Co-sensitized patients benefit more from Pooideae AIT when dominant, but no head-to-head clinical trials directly compare Pooideae versus Chloridoideae AIT products in patients with dual sensitization. Nevertheless, an AIT based solely on *Phleum pratense* pollen is unlikely to be optimized for *Cynodon* pollen monosensitization, and its efficacy for Chloridoideae-specific allergy remains unproven based on available data. It is suggested that subfamily-specific AIT formulations may be necessary for optimal results in a few European patients [47,173,183,184,185].

A more specific assessment of grass pollen sensitization profiles across multiple climatic regions in Europe may help precisely identify the relevant grass pollen and achieve the most efficacious AIT for an individual patient [118,173].

In the EU, AIT formulations are classified as medicinal products under Directive 2001/83/EC, which generally requires marketing authorization to demonstrate quality, safety, and efficacy. AIT products listed by the Paul-Ehrlich-Institut (PEI), the national authority for allergen products in Germany and a prominent player in European allergen harmonization efforts, may be administered subcutaneously or sublingually [142]. Injectable AIT products for Pooideae grass pollen allergy are typically authorized nationally in Europe. Some subcutaneous immunotherapy (SCIT) products are native grass pollen allergens (non-modified) as depot formulations, adsorbed onto aluminum hydroxide. In contrast, others are allergoids (chemically modified allergens) polymerized with formaldehyde or glutaraldehyde. The first ones are available as depot products, adsorbed onto aluminum hydroxide. Those modified with glutaraldehyde are either available as a depot with tyrosine (L-tyrosine adsorbate), with or without an immunostimulant adjuvant (monophosphoryl lipid A as TLR4 agonist), or adsorbed onto aluminum hydroxide [186]. Two Pooideae grass pollen orodispersible tablets are EMA-authorized sublingual AIT (SLIT) products: a five-grass mixture sublingual tablet (Oralair^®^; Stallergenes Greer, Antony, France) containing natural pollen allergens from five of the major Pooideae grass species (*Phleum pratense*, *Anthoxanthum odoratum*, *Dactylis glomerata*, *Lolium perenne*, *Poa pratensis*) and a single-grass sublingual tablet/oral lyophilizate (Grazax^®^; ALK-Abelló, Hørsholm, Denmark) containing standardized allergen extract of Timothy grass (*Phleum pratense*) pollen. This single-grass sublingual tablet contains an average Phl p 5 content of 6 μg per lyophilizate, as determined by European Pharmacopoeia (Ph. Eur.) methods and as stated in the summary of product characteristics (SmPC). Scientific literature often reports higher equivalent values of approximately 10–15 μg of Phl p 5, likely due to variations in immunoassay techniques. For the five-grass mixture sublingual tablet, the SmPCs do not specify Phl p 5 content using Ph. Eur. methods. However, published data indicate that it contains approximately 20–25 μg of Phl p 5 or group 5 major allergens, as determined by in-house or independent assays [187,188]. Several other SLIT products, such as carbamylated monomeric allergoid tablets and liquid-based SLIT formulations delivered as drops from dropper bottles, or SLIT products based on a pump or spray mechanism for delivery, may be supplied under older local approvals in specific contexts or as NPPs pursuant to Article 5(1) of Directive 2001/83/EC. Availability greatly varies by country due to regulatory heterogeneity. For regionally prevalent and lower-prevalence grass pollen allergens, such as those from *Cynodon dactylon*, AIT products are supplied exclusively as NPPs, typically as liquid solutions in metered-dose devices, which may be permissible under the broader European framework set out in the above-mentioned Article 5(1). In several European regions with high exposure to *Cynodon* pollen, this exemption may support individual patient needs, because authorized alternatives are currently unavailable. Although FDA-regulated Bermuda grass pollen extracts are used for SCIT in the United States, no EMA-approved standardized SCIT product exists specifically for *Cynodon dactylon* grass pollen allergy [158,186,189,190,191].

There is reason for optimism in the evolving regulatory landscape within Europe, as a new EMA guideline on AIT (EMA/161669/2025) becomes legally effective on 1 January 2026 and provides tailored recommendations to facilitate marketing authorizations for products targeting lower-prevalence allergens, including those with limited patient populations [192].

Finally, it is important to mention that clinical indications for AIT have been well documented for respiratory allergy in the presence of rhinitis and/or allergic asthma to grass pollen, with absolute contraindications including severe asthma, active malignancy or active uncontrolled systemic autoimmune conditions, along with initiation of treatment during pregnancy. In selected cases, with individual risk-benefit assessment, grass pollen AIT can also be initiated in the presence of relative contraindications, such as primary and secondary immunodeficiencies. For instance, Pooideae grass tablet SLIT is considered efficacious, safe, and well-tolerated in HIV-positive patients on highly active antiretroviral therapy [193,194,195].

Inborn errors of immunity (IEIs) with atopic phenotypes represent a growing and complex subset of over 50 monogenic disorders that manifest primarily through severe and multiple allergic symptoms. In hyper-IgE syndromes and IEIs with significant antibody deficiencies, AIT may have limited benefits due to their complex immune dysregulation. For these patients, clinical immunologists often pivot away from AIT and toward biological therapies, as these methods bypass the need for the patient’s immune system to retrain itself [196,197,198]. Even the in vitro diagnosis can be challenging in IEIs. In patients with antibody deficiencies such as common variable immunodeficiency (CVID), the majority present with undetectable total and ssIgE levels, with IgE immunoassays returning negative values. Therefore, allergy assessment via serology is not useful in such clinical scenarios. In CVID patients with a personal history suggestive of grass pollen allergic rhinitis, diagnostic nasal provocation may be suggested [199,200,201]. Regarding high total serum IgE concentrations, levels up to 3000 kU/L do not affect the performance of modern singleplex and multiplex IgE assays. In specialized evaluations conducted for regulatory approval, spiking experiments with extremely high total serum IgE levels (e.g., 3000 kU/L and 10,000 kU/L) have occasionally yielded discordant results. The high-dose hook effect, also known as the prozone effect in particular immunoassay contexts, occurs when excessive concentrations of ssIgE in undiluted samples overwhelm the immunoassay’s binding capacity, leading to underestimation or discordant quantification of ssIgE due to incomplete complex formation [133,202].

Several limitations are evident. The diversity of the European population is underrepresented because data are typically collected by specialized clinics in a restricted number of countries. The limited availability of multiplex immunoassays, nasal provocation tests, and standardized clinical symptom scores across Europe introduces methodological constraints, while singleplex tests targeting a narrow range of grass pollen components may miss some relevant IgE sensitization profiles. The potential influence of CCDs also deserves consideration. Addressing these limitations in future large-scale European studies is crucial to strengthening the evidence base on CRD in grass pollen allergy.

## 6. Conclusions

Molecular grass pollen allergens used in CRD represent the basis for precision allergy diagnostics in Europe [1,31,58,85,203]. Such an important strategy is currently recommended in clinical settings in the European Union, particularly in cases with multiple positive results from in vivo and in vitro allergy tests using natural grass pollen wae, to discriminate between genuine sensitization and cross-reactivity (Table 5). This approach also enables clinicians to identify specific risks and optimize AIT.

Knowledge of molecular allergen biomarkers and updated diagnostic algorithms is also essential in allergy clinical practice for optimizing AIT. Additional biomarkers are required to monitor AIT effectiveness and identify non-responders more efficiently.

## Figures and Tables

**Table 2:** Overview of the allergenic molecules used in commercially available IgE immunoassays from pollen of *Phleum pratense* and *Cynodon dactylon*, as clinically relevant European Pooideae and Chloridoideae grasses [1,123].

Allergen	IgE Sensitization Prevalence	Biochemical Name, Function, Group	Molecular Weight (kDa)
Phl p 1 Timothy grass	83–95%	β-expansin (CCD-bearing)	27
Phl p 2 Timothy grass	55–65%	expansin-like domain (Grass group 2)	10–12
Phl p 4 Timothy grass	70–75%	berberine bridge enzyme (CCD-bearing)	55
Phl p 5 Timothy grass	50–95%	ribonuclease (Grass group 5)	29–32
Phl p 6 Timothy grass	44–75%	acidic peptide (Grass group 6)	11
Phl p 7 Timothy grass	7–10%	polcalcin (calcium-binding protein)	6
Phl p 11 Timothy grass	32–43%	Ole e 1-related trypsin inhibitor protein	20
Phl p 12 Timothy grass	15%	profilin (actin-binding protein)	14
Cyn d 1 Bermuda grass	76–100%	β-expansin (CCD-bearing)	32

Note: The abbreviation for a molecular allergen consists of: genus abbreviation (first three letters of the scientific genus name of the source organism, e.g., “Phl” for *Phleum*), species abbreviation (first letter of the species name, e.g., “p” for *pratense*) and allergen number (a sequential integer indicating the order of discovery or characterization, e.g., “1” for the first identified allergen from that source).

**Table 3:** Molecular allergen components of grass pollen, which are used in commercially available singleplex and multiparameter IgE immunoassays in Europe.

Allergen Component, Grass Common Name	Protein, Grass Latin Name	Code	Singleplex Assay	Multiparameter Assay
rPhl p 1 Timothy grass ^	β-expansin *Phleum pratense*	g205	ImmunoCAP^®^ FEIA	DPA-Dx * P1, SE1, ESEP
nCyn d 1 Bermuda grass	β-expansin *Cynodon dactylon*	g216	ImmunoCAP^®^ FEIA	
rCyn d 1 Bermuda grass	β-expansin *Cynodon dactylon*	g216		DPA-Dx * ESEP
rPhl p 2 Timothy grass	expansin-like domain *Phleum pratense*	g206	ImmunoCAP^®^ FEIA	
nPhl p 4 Timothy grass	oxidoreductase *Phleum pratense*	g208	ImmunoCAP^®^ FEIA	
rPhl p 5 Timothy grass ^	ribonuclease *Phleum pratense*	g215	ImmunoCAP^®^ FEIA	DPA-Dx * P1, SE1, ESEP
rPhl p 6 Timothy grass	acidic peptide *Phleum pratense*	g209	ImmunoCAP^®^ FEIA	
rPhl p 7 Timothy grass ^^	polcalcin *Phleum pratense*	g210	ImmunoCAP^®^ FEIA	DPA-Dx * P1, SE1, ESEP
rPhl p 11 Timothy grass	trypsin inhibitor *Phleum pratense*	g211	ImmunoCAP^®^ FEIA	
rPhl p 12 Timothy grass ^^	profilin *Phleum pratense*	g212	ImmunoCAP^®^ FEIA	DPA-Dx * P1, SE1, ESEP

Note: FEIA, fluorescence enzyme immunoassay; n, natural purified; r, CCD-free recombinant; ^ individual (g205 and g205) or together rPhl p 1 and rPhl p 5b (g213) in ImmunoCAP^®^ FEIA; ^^ individual (g210 and g212) or together rPhl p 7 and rPhl p 12 (g214) in ImmunoCAP^®^ FEIA; * DPA-Dx, Euroline™ line blot immunoassay defined partial allergens diagnostics, P1 (Pollen 1), SE1 (Southern Europe 1), customized ESEP (Euroline Southern European Pollen Profile) [140,145,146].

**Table 4:** Molecular allergen components of grass pollen, which are used in commercially available multiplex IgE immunoassays in Europe.

Allergen Component, Grass Common Name	Protein, Grass Latin Name	Code	Microarray Assay	Macroarray Assay
rPhl p 1 Timothy grass	β-expansin *Phleum pratense*	g205	ImmunoCAP^®^ ISAC E112i	ALEX3^®^
nCyn d 1 Bermuda grass	β-expansin *Cynodon dactylon*	g216	ImmunoCAP^®^ ISAC E112i	
rCyn d 1 Bermuda grass	β-expansin *Cynodon dactylon*	g216		ALEX3^®^
nZea m 1 maize pollen	β-expansin *Zea mays*	g217		ALEX3^®^
rPhl p 2 Timothy grass	expansin-like domain *Phleum pratense*	g206	ImmunoCAP^®^ ISAC E112i	ALEX3^®^
nPhl p 4 Timothy grass	oxidoreductase *Phleum pratense*	g208	ImmunoCAP^®^ ISAC E112i	
rPhl p 5 Timothy grass *	ribonuclease *Phleum pratense*	g215	ImmunoCAP^®^ ISAC E112i	ALEX3^®^
rPhl p 6 Timothy grass	acidic peptide *Phleum pratense*	g209	ImmunoCAP^®^ ISAC E112i	ALEX3^®^
rPhl p 7 Timothy grass	polcalcin *Phleum pratense*	g210	ImmunoCAP^®^ ISAC E112i	ALEX3^®^
rPhl p 11 Timothy grass	trypsin inhibitor *Phleum pratense*	g211	ImmunoCAP^®^ ISAC E112i	
rPhl p 12 Timothy grass	profilin *Phleum pratense*	g212	ImmunoCAP^®^ ISAC E112i	ALEX3^®^

Note: Immuno CAP^®^ ISAC™ E112i is an advanced iteration of the technology of ISAC™ = Immuno Solid-phase Allergen Chip; ALEX3^®^ is an improved successor product of ALEX^®^ = Allergy Xplorer ELISA-based macroarray immunoassay; it integrates a CCD inhibitor; n, natural purified; r, CCD-free recombinant; * rPhl p 5b in ImmunoCAP^®^ ISAC E112i or rPhl p 5.0101 in ALEX3^®^ [124,140].

**Table 5:** Summary of key molecular allergen biomarkers for IgE immunoassays and tips for clinical implications in grass pollen allergy in Europe.

Biomarker	Protein Type	Value as Allergen Component for Molecular Diagnosis and Clinical Associations
rPhl p 1	β-expansin	major allergen biomarker specific for primary IgE sensitization to grass pollen (Poaceae family), its native form is often the initiator molecule in grass pollen allergy
rCyn d 1	β-expansin	major allergen biomarker specific for primary IgE sensitization to pollen of warm temperate/subtropical grasses of the Chloridoideae subfamily
rPhl p 5	ribonuclease	major allergen biomarker specific for primary IgE sensitization to pollen of temperate grasses of the Pooideae subfamily
rPhl p 2	expansin-like domain	biomarker that confirms genuine IgE sensitization to grass pollen, predominant allergen biomarker in subpollen particles from grasses associated with the risk of thunderstorm-related asthma
rPhl p 7	polcalcin	minor panallergen biomarker of cross-reactivity among pollen grains from trees, grasses, weeds, potential biomarker for risk of severe disease and multimorbidity
rPhl p 12	profilin	minor panallergen biomarker of pollen-food cross-reactivity, biomarker for the risk of oral allergy syndrome

Note: ssIgE to rPhl p 1 but not to rCyn d 1 supports the diagnosis of Timothy/Pooideae grass pollen primary IgE sensitization. In contrast, ssIgE to rCyn d 1 but not to rPhl p 1 supports the diagnosis of Bermuda/Chloridoideae grass pollen primary IgE sensitization, if ssIE to rPhl p 5, rPhl p 6, or rPhl p 2 are also not detected. In fewer cases with no detectible ssIgE to rPhl p1 and rCyn d 1, the detection of ssIgE to at least one of the biomarkers rPhl p 5, rPhl p 6, or rPhl p 2 may confirm the diagnosis of Pooideae pollen genuine IgE sensitization. In case of ssIgE to both rPhl p 1 and rCyn d 1, if values of ssIgE to rPhl p 1 > rCyn d 1 and ssIgE antibodies are detected against at least one of the biomarkers rPhl p 5, rPhl p 6, or rPhl p 2, then the diagnosis of Timothy/Pooideae grass pollen primary IgE sensitization is supported with significant probability. If ssIgE to both primary biomarkers rPhl p 1 and rCyn d 1 and values of ssIgE to rCyn d 1 > rPhl p 1, along with detection of ssIgE antibodies against at least one secondary diagnostic biomarker rPhl p 5, rPhl p 6, or rPhl p 2, then the diagnosis of Timothy and Bermuda pollen IgE double sensitization is evident [1,31,52,56].

## Data Availability

No new data were created or analyzed in this study.
